# Barriers, facilitators and potential solutions to implementing Kiddie Schedule for Affective Disorder and Schizophrenia (KSADS) screening tool at Muhimbili National Hospital in Dar es Salaam, Tanzania

**DOI:** 10.1371/journal.pone.0323502

**Published:** 2025-05-09

**Authors:** Leonida Isdory Ngongi, Christopher Fittipaldi Akiba, Mrema Noel Kilonzo, Anna Agape Minja, Charles Onesphor Komba, Mwajabu Rashidi Mbaga, Anna Celestini Msafiri, Lusajo Joel Kajula, Sylvia Florence Kaaya, Brian Wells Pence, Bradley Neil Gaynes

**Affiliations:** 1 Muhimbili National Hospital, Dar es Salaam, Tanzania; 2 RTI International, Research Triangle Park, North Carolina, United States of America; 3 Muhimbili University of Health and Allied Science, Dar es Salaam, Tanzania; 4 Gillings School of Global and Public Health, University of North Carolina At Chapel Hill, Chapel Hill, North Carolina, United States of America; University of Kansas School of Medicine Wichita, UNITED STATES OF AMERICA

## Abstract

**Background:**

Attention Deficit Hyperactivity Disorder (ADHD) affects 5% of adolescents globally. ADHD increases the child’s risk for adverse outcomes, including school failure, juvenile delinquency, substance abuse, and increased sexual risk behaviors. ADHD can be diagnosed in children using the Kiddie Schedule for Affective Disorders and Schizophrenia (KSADS). Semi-structured or structured diagnostic interviews, such as the KSADS-Present and Lifetime (PL) version by Kaufman and colleagues, are the gold standard in diagnosing psychiatric disorders like ADHD. Nevertheless, KSADS-PL is not used in routine clinical practice in Tanzania. There is no research exploring barriers and facilitators to use of KSADS-PL in resource limited areas including Tanzania. The study aimed to uncover barriers, facilitators and possible solutions related to psychiatric care providers’ routine use KSADS-PL at the Muhimbili National Hospital (MNH) in Dar-es-Salaam, Tanzania.

**Methods:**

Between July and October 2019, we conducted semi-structured interviews that focused on providers’ perceptions of facilitators, barriers, and solutions regarding KSADS-PL integration into routine clinical practice, data were analyzed data using a qualitative thematic approach informed by the Consolidated Framework for Implementation Research (CFIR).

**Results:**

Limited knowledge and lack of training about KSADS-PL represented the most mentioned perceived barrier for providers. Some providers reported inadequacy of both human and material resources, high workload, and limited physical space at the clinic. Facilitators included readiness for KSADS-PL implementation, and providers’ desires for uniform and standardized ways of detecting ADHD. Suggested solutions included involving hospital leaders, support for provider training, increasing staff, making KSADS-PL tools readily available, utilizing an online version of the tool, creating departmental standards, maximizing space at the clinic, and reorganizing clinic flow.

**Conclusion:**

Findings suggest a need for innovative implementation science solutions such as multifaceted educational strategies focusing on ongoing trainings and supervisions to increase clinical knowledge, reorganizing clinic flow to increase the quality and duration of patient‑provider interaction, as well as role shifting and other planning strategies that may address barriers like understaffing.

## Introduction

A mental health treatment gap persists for children and adolescents in sub-Saharan Africa which have limited treatment capacity relative to high income countries [1]. The World Health Organization’s Mental Health Gap Action Plan and the push for mental health to be included in the Millennium Development Goals have raised the profile of child mental health but comparatively few studies address the importance of screening tools for mental health conditions in these settings [1]. In high-income countries, attention deficit hyperactivity disorder (ADHD) is one of the most prevalent and highly investigated conditions in child and adolescent mental health [2].

Studies focused on sub-Saharan Africa highlight the prevalence of ADHD to range from 5.5% to 8.7% among school children in Congo [3]. A more recent meta-analysis by Ayano and colleagues in 2020 focusing on studies conducted in the African setting, showed a pooled prevalence of ADHD in children and adolescents to be 7.47% [4]. The disorder places the child at increased risk for school failure and dropout, juvenile delinquency, criminality, substance abuse, sexual risk behaviors, and, as a possible consequence, HIV and other sextually transmitted infections [5,6]. According to the Global Burden of Disease, ADHD is responsible for 500,000 disability-adjusted life years (DALYs), 0.2% of the burden of all mental health disorders [7].

Given the prevalence and impact of ADHD, strategies to integrate ADHD screening in general medical care are needed. One tool that has been validated for identification of ADHD in African settings, the Kiddie Schedule for Affective Disorder and Schizophrenia-Present and Life time version (KSADS-PL). Despite its efficacy in rural Kenya, there is no known research exploring challenges on using KSADS-PL in routine practice in similar resource limited settings throughout the region [8]. Therefore, there is a need to identify barriers and possible solutions informing strategies to improve the uptake of the KSADS-PL in routine clinical practice among psychiatric settings that treat children at risk of ADHD. The current study aimed to describe the facilitators, barriers and their potential solutions to the uptake of the KSADS-PL at Muhimbili National Hospital (MNH) in Dar es Salaam, Tanzania.

## Materials and methods

### Study setting

The current qualitative study is part of the formative phase of a subsequent implementation trial testing the effectiveness of a multifaceted implementation strategy to increase the acceptability and feasibility of the KSADS-PL. The present study specifically explored the possibilities of introducing KSADS-PL for screening and diagnosing ADHD at MNH’s child and adolescent clinic, where children throughout Tanzania are referred for specialized mental healthcare. Providers in the current study used KSADS-PL’s ADHD screening items and ADHD diagnostic supplements only, taking into consideration that there were existing routine ways of screening for other child and adolescent psychiatric disorders at the clinic. During the formative phase of the study, the clinic had been operating one day per week with an average of 28–32 patients per day. The ratio of patient to provider in every clinic day averages 6–8 patients per provider depending on provider’s cadre. A recent push at the government level, as well as plans for developing and piloting, an electronic health record (Mental Health Information Reporting Assistant) for evidence-based assessments at MNH has increased focus on improving the quality of specialized mental healthcare, creating an opportunity for the present study [9].

### Study design

We used a qualitative approach supported by the Consolidated Framework of Implementation Research, CFIR [10]. The CFIR facilitates a systematic assessment of multilevel domains and constructs to identify factors that might influence intervention implementation.

### Study participants

Participants included providers at the Department of Psychiatry and Mental Health at MNH. We included providers from different cadres in the department attending to children and adolescents including nurses, social workers, occupational therapists, general practitioners, residents in master of medicine in psychiatry, residents in clinical psychology, and psychiatrists.

### Data collection methods and tools

Our data collection plan included: four (4) key-informant interviews (KII) with the Director of Medical Services, the Head of the Psychiatry Department, and a clinical psychologist and psychiatrist in leadership roles at the clinic. Twenty (20) in-depth interviews (IDI) were planned with psychiatrists (n = 8), clinic nurses (n = 2), clinical psychologists (n = 4), occupational therapists (n = 2), social workers (2) residents in psychology and psychiatry (n = 2). Due to time constraints and the practice of limited interactions following COVID-19 protocols, we only managed complete eleven (11) out of the 20 IDIs.

Semi-structured interview guides framed questions and relevant probes (S2 File). Given the background of mental health care training in Tanzania, the semi-structured guides elicited providers’ experiences assessing ADHD, their thoughts when given a description of the KSADS-PL, their perspectives on improving screening and diagnostic capacity for ADHD at MNH, their thoughts on the KSADS-PL’s integration into routine services within the child and adolescent clinic, barriers, and suggestions on how integration might be streamlined. The interviews were conducted by one senior qualitative researcher (AAM), in-person, and on site at MNH. AAM is a social scientist with post-graduate qualifications and previous experience in conducting interviews in mental health care settings (S1 File).

### Data management and analysis

All interviews were conducted in Swahili, audio-recorded, and supplemented with field notes taken during or just after the interviews. Audio recordings were transcribed and translated into English using a one-step approach (i.e., Swahili sentences within the interview were transcribed directly into English by a bilingual transcriptionist), while field notes were expanded and used to write summaries that fitted within the transcripts.

Thematic analysis was facilitated by Dedoose Version 9.0.54 and aided by the CFIR constructs. Naeem and colleagues (2023) describe thematic analysis as a process guiding researchers’ selection of keywords and quotations, coding, identification of themes, interpretation, and the development of a conceptual model [11]. Accordingly, two senior qualitative study staff developed a codebook originating from questions on the interview guides and additional codes were added as themes emerged. We used both inductive and deductive approaches to identify facilitators or barriers to KSADS-PL utilization. Deductive codes were created before coding began and were generated them from the interview guide. For example, codes like ‘barriers to KSADS-PL uptake’ were created from the question “What might be the possible barriers or things that hinder the implementation process for K-SADS-PL use at your clinic?”.

Inductive codes were generated once coding began, emanating from the data itself. For example, we decided we needed a code for “perceived increased workload” due to providers describing fears regarding increased workload following KSADS-PL integration. Coded themes were further developed by coding summaries and matrices to better understand mental health providers’ readiness towards the introducing KSADS-PL use at the clinic. Table 1 below summarizes codes and subcodes which were addressed in the current study.

**Table 1:** Barriers, facilitators and potential solutions to implementing KSADS at child and adolescent clinic, MNH.

THEMES	CODES	SUBCODES
Barriers	Inadequacy of both human and material resources	Limited number of providers at the clinic Unavailability of the tool Limited number of hard copies of tools Limited physical space
	Providers’ perception on the use of standardized tool like the KSADS-PL	perceived increased workload due to KSADS-PL integration Limited provider knowledge about KSADS-PL tool Lack of orientation/training about KSADS-PL tool Lack of on-job training/ongoing supervision
Facilitators	Facilitators to reinforce the use of the KSADS-PL	Providers’ readiness and motivation to help children
		Providers’ motivation on use of KSADS-PL as a uniform way of assessing clients
		Restructuring of clinic’s daily operations
Potential solutions	Recommended solutions to address identified barriers	Orienting/training providers before use of KSADS-PL Ongoing supervision at the clinic Involvement of hospital management and other potential stakeholders Maximizing space at the clinic Availability of both human and non-human resources Use of electronic version of KSADS-PL instead of hard copies Role shifting Provision of extra duty allowances

### Ethical considerations

The Muhimbili University of Health and Allied Sciences (MUHAS) Senate Research and Publications Committee review board approved this pilot study (Ref: No. 282/298/01.C/) and MNH’s research unit granted permission to conduct the study. Study participants provided written informed consent. All participants gave consent for interview transcripts to be published. The transcripts do not contain any potentially identifying information.

## Results

We were unable to complete four of the planned key informant interviews with stakeholders at the clinic, and we only completed 11 out of the planned 20 in-depth interviews due to the COVID-19 pandemic. In-depth interviews included a nursing block manager (n = 1), psychiatrists (n = 4), and a clinical psychologist (n = 1), psychiatry resident trainee (n = 1), a registrar (n = 1), social worker (n = 1), nurse (n = 1), and occupational therapist (n = 1).

### Overview of key findings

All providers welcomed the use of KSADS-PL and highlighted its utility as a screening and diagnostic tool for ADHD. All of the interviewed providers mentioned that they regularly treat children they presume to have ADHD and described a need for improving screening and diagnosis.

Providers’ responses reported a number of barriers, mostly relating to inadequacy of both human and material resources like the limited number of providers at the clinic, the unavailability of the tool, high provider workload, and limited space at the clinic.

Despite these challenges, providers’ readiness and motivation to help children and adolescents at the clinic were highlighted as facilitators of KSADS-PL implementation.

Providers recommended possible solutions to the identified barriers like training and ongoing supervision, making the tool readily available, adding the KSADS-PL be into the departmental standard operating procedures, and utilizing the electronic version of the tool. The following section explains in detail, facilitators, barriers, and solutions to implementing use of KSADS-PL with their categorization into CFIR domains.

### Facilitators to implementing use of KSADS-PL

#### Characteristics of the individual.

Our study found that most providers understood the importance of having a formalized way of screening and diagnosing ADHD and other mental disorders at the clinic. Most expressed their motivation in helping children referred to the clinic:

“*What motivate us is when you provide service to a patient and the patient gets better…. Also. [the KSADS-PL] will be more efficient and different from the way we do it now because we use DSM-5 and taking clinical symptoms so if we have a tool [for ADHD] it will bring uniformity*.” Psychiatrist

In the following quote, a psychiatrist similarly emphasized how introducing a uniform method of screening for ADHD would help to more properly identify patients.

“*It will be easier and since this tool will be already validated, it will bring uniformity among us, and since it is made special for diagnosing ADHD it becomes easier when you have a checklist because you will be assessing only specific indicators.”* Psychiatrist

#### Intervention characteristics.

Providers reported that KSADS-PL seemed feasible and easy to use. This level of perceived feasibility relates directly to the CFIR construct of complexity, focused on the length and breadth of an intervention. Participants described how introducing a straightforward tool would help them to identify ADHD and other mental disorders in a systematic way.

*“It would be easier to use the tool if you compare with others, it is because of the way it is, it is summarized and explained well the symptoms.”* General Practitioner/Medical Doctor

“*Services are improved [with such a tool] because you attend many children for a short time and get important things.”* Clinical psychologist

The quotes above highlight key aspects of the KSADS-PL like its simplicity and brevity. Both represented valued intervention characteristics for participants given the high volume of patients who present during clinic hours.

#### Inner settings.

Providers explained how a recent clinic reorganization would facilitate KSADS-PL uptake. In the quote below, a clinical psychologist noted that clinic flow had recently been reorganized, allowing for improvements regarding the flow of patients including triaging children, and designating specific days for assessments and other interventions. Interviewees felt like these changes might facilitate uptake of KSADS-PL by reducing providers’ workloads, improving time spent with patients, and shortening the duration of wait time during patients’ visits.

“*…. we have shifted the clinic at the children building, we want any patient who comes for the first time should go through screening or diagnosis, then after that results will be discussed with a larger team…*.” Clinical psychologist

The quote highlights structural and operational changes made at the clinic to facilitate implementation of a tool like the KSADS-PL.

#### Barriers and possible solutions to implementing the KSADS-PL.

Providers reported a number of challenges that might hinder KSADS-PL implementation. Most of the reported barriers related to the CFIR domains of characteristics of the individual, the outer setting, characteristics of the intervention, and inner setting. The most salient barriers reported by participants included limited provider knowledge about the KSADS-PL, perceived increased workload due to KSADS-PL integration, limited number of staff, and anticipated inadequate material resources like insufficient copies of the tool. Interviewees recommended possible solutions to the identified barriers as well. We describe each following participants’ descriptions of barriers throughout this section.

#### Characteristics of individuals.

Because the KSADS-PL represented a novel diagnostic tool, interviewees highlighted their lack of familiarity as a barrier that could be addressed through training. They reported how being unfamiliar with the KSADS-PL might increase the duration of patient-provider interactions:

“*We are not familiar with the checklist therefore the time will not be the same with normal follow up…If I use KSADS-PL in the first day, first I need to take the history, which I can spend one hour and then I use this checklist…If I would practice or implement it for a long time, people would already put it in their mind.*” Psychiatrist

“*Maybe because I am not familiar with it so it might be difficult in asking direct questions directly to a parent, so I need more time to familiarize myself to it.*” Occupational therapist

Both providers reported how their lack of familiarity might pose challenges regarding the KSADS-PL, as the psychiatrist reported it would take more time and the occupational therapist described challenges related to using a tool they were not yet familiar with.

In addition to training in general, providers suggested orienting and training all providers at the department, rather than select a few. Providers described how new interns frequently rotate through the department alongside residents in their postgraduate psychiatry and clinical psychology studies. Because these providers transfer across departments at the hospital, providers highlighted the importance of training them in addition to more regular staff so that any client visiting the clinic could benefit from an assessment guided by the KSADS-PL.

“*I think the main issue is for everyone to be trained…if we have specific tools like KSADS-PL we have to train them how to use the tools and the tools should be accessible.”* Psychiatry Resident Doctor

However, other staff worried that the training of all providers may prove challenging in the face of busy clinic service. While they agreed that the clinic could benefit from all providers sharing in their ability to screen patients using the KSADS-PL, they found it challenging to envision successfully training all providers.

#### Inner setting.

Inadequate resources required to introduce the KSADS-PL represented a common barrier described by the providers. Specifically, an inadequate number of providers and a lack of physical space were reported as the most salient inner setting barriers. Providers perceived that introducing the KSADS-PL, in an already understaffed and overworked environment, would create additional work for the already overstretched mental health providers.

Most providers insisted that introducing KSADS-PL will require training staff of all cadres who see patients at the clinic including nurses, social workers, and occupational therapists. They perceived that if the assessments were shifted to other staff apart from psychiatrist and clinical psychologist, it might reduce the workload overall. Their opinion is represented by the quote below:

”D*iagnosis can be done by even a nurse because a nurse is the first person to see the patients before a doctor. It will simplify the work especially for our country because there are few specialists.”* Psychiatrist

Other providers emphasized the inadequacy of physical space at MNH as a barrier to KSADS-PL utilization:

“*A good environment can help to provide servi, for example the place where the present clinic is located is very small.*” Clinical psychologist

Recommendations to address these barriers included hospital management for maximizing clinic space, adding more clinic days and working shifts, and making tools available or accessible online:

“*We have only one clinic day in a week, so children are many and the space is small, so it becomes a challenge to attend them. It will be better if there will be good arrangement like what is been done to the adult clinic because we have four units, so it should be the same to children because the space at pediatric clinic is very small…*” Psychiatrist

“*It will require some money for things like producing copies or printing…I suggest that it will be better if it will be available in the [electronic medical] system to make it accessible to everyone.”* Psychiatrist

Providers described a lack of staff familiarity with the tool as a structural barrier to its uptake. They suggested a structural solution by codifying the KSADS-PL as routine practice within the department:

“*…people are not familiar with the tool. And familiarization means departmentalized that means should be entered into department to be as a routine….*” Psychiatrist

As illustrated by the quotes above, providers suggested reorganizing clinic operations, increasing clinic space, and using electronic versions of the tool to address the identified barriers.

#### Outer setting.

Participants raised concerns over the cost of trainings associated with the introduction of the KSADS-PL. In response, they mentioned involving outside organizations (both governmental and non-governmental) as solutions to some of the barriers. For example, a social worker mentioned the need for the Ministry of Health and Ministry of Education to support not only provider training, but also ADHD awareness campaigns, to facilitate early identification and screening programs. The two quotes below narrated these recommendations, while the second quote alluded to an important additional barrier:

“*If they will be able to provide support in the coordination between us and school or with Ministry of Education…because we are the health experts so they can empower us to know this tool very well either by giving us on job training, short courses so that we can understand and identify children with special needs and be able to teach the community and families in general.*” Social worker

“*Maybe now we have these kinds of children in the community and we missed them because people don’t understand these children, they don’t know where to send them, they don’t know what to do. Therefore, everyone has her/his own views about these children. If we could get that chance of being educated and empowered enough, we can enter to the community, and schools, and be able to empower other people and teachers [to identify children and adolescents with ADHD], everything would be going on well.*” Social worker

The above quote describes the importance of training and its impact on not only provider education on adolescent ADHD but an added benefit to community awareness. Unlike our other participants, the social worker above additionally highlighted how “everyone has her/his own views about these children.” This suggests that stigmatizing beliefs about adolescents with ADHD or perhaps other mental health disorders may crop up in the absence of community education of psychiatric conditions. The implications of this and our other findings are highlighted further in our discussion section.

## Discussion

The current study intended to add to the literature on the use of the KSADS-PL as an evidence-based assessment tool for screening and diagnosing ADHD among children and adolescents in Tanzania. We used CFIR domains and constructs to categorize facilitators, barriers, and their solutions regarding the implementation of the KSADS-PL. In our sample, we found that all the interviewed providers showed a willingness to use KSADS-PL. Providers conveyed their motivation to help children they routinely see at the clinic, and they felt a level of satisfaction seeing their clients improve after engaging in treatment. Most providers mentioned about their current use of the DSM-5 as a diagnostic guideline, highlighting their knowledge on ADHD diagnostic criteria; because the KSADS-PL represents a DSM-5 based tool, MNH providers would likely possess some knowledge regarding the tool’s items [12].

To address barriers related to costs associated with mass producing paper copies of the KSADS-PL, some of providers suggested the use of electronic version. Their suggestion can be strengthened by findings from Townsend and colleagues (2020), who focused on development of computerized versions of KSADS (KSADS-COMP). They concluded that use of clinician-administered KSADS-COMP indicates utility in clinical settings, as it can also shorten administration due to its ability to score automatically [13]. Regarding additional barriers described in our results section, it is likely not realistic to suggest that governing bodies in resource-constrained health systems provide additional funds to hospitals to increase staffing, material resources, and physical space [14]. With regard to the literature base, the implications of these results may be best addressed through innovative implementation strategies to address barriers within the inner setting like task-shifting, champions, train-the-trainer models, and organizational coaching [15,16].

Our findings reflected similar conclusions from the implementation and mental health literature, indicating how a barrier like limited knowledge can be addressed by provision of training to providers; and then, continuing to build their capacity through provision of ongoing technical assistance [16–18]. Therefore, initial and ongoing provider trainings, provision of technical assistance, and supervision may facilitate uptake of the KSADS-PL in this setting. Precedence for similar strategies exist within the region. For example, in a study done in Tanzania by Mwendwa and colleagues that aimed to improve the human resource management (HRM) function at district and health facility level through the Support, Train and Empower Managers (STEM) project to foster the capacity of managers to support and supervise their staff in Tanzania [19]. Their findings showed how training and supervision support the implementation of STEM as an evidence-based intervention, and ultimately enabled an environment for management to support staff and improve their morale and retention. Another study from Malawi concluded that an mhGAP training package could be delivered to large numbers of non-specialist health care workers, and was successful at improving their knowledge, confidence, and case detection rate of mental health disorders. Using a “train the trainers” model may also ensure that the knowledge and skills of district mental health teams are updated and strengthened, remaining as a resource within the district [20].

Inadequacy of both human and material resources such as staff and medications, lack of mental health training, lack of physical space, and a lack of coordination within healthcare systems have been highlighted as barriers to implementing evidence-based health interventions in low and middle-income countries like Tanzania [21]. Providers reported that most of the health facilities in Tanzania experience understaffing and added that the majority lack trained mental health providers. “Task shifting” may be employed to implement evidence-based interventions like use of KSADS-PL. Task shifting involves the use of non-specialized staff to perform duties of specialized staff, and has been widely adopted in sub-Saharan Africa across various fields of health [22,23]. Suggestions from our participants aligned with these concepts when they described how providers from all cadres including nurses, social workers, occupational therapists, general practitioners’ residents, clinical psychologists, and psychiatrists could benefit from the ability to screen and diagnose ADHD using the KSADS-PL.

While task shifting in theory represents a solution to the lack of trained mental health specialists, recent work from the region describes how task shifting alone may not adequately address inner and outer setting barriers. Akiba et al. (2023) described how chronic disease clinic provider fidelity to administering a newly integrated depression screening tool varied based on several factors related to clinic leadership and implementation climate [24]. These results suggest that task shifting might succeed best when paired with additional implementation strategies aimed at addressing additional health systems barriers. In a recent review of task shifted interventions throughout sub-Saharan Africa, Okoroafor and Christmals (2023) concluded that task shifting represents a viable solution to shortages of medical specialists (e.g., psychiatrists) [25]. However, the authors caution that due to the complexities of health systems in the region, needs assessments to better understand implementation contexts should precede any additional strategies. Okoroafor and Christmas (2023) note that once barriers are understood, strategies like workforce capacity building and provision of necessary implementation tools (e.g., job aids) are likely to follow. These suggestions align closely with our study results where providers describe the importance of adequate and ongoing training to address turnover, as well as the availability of the KSADS-PL.

While our results describe unattended barriers that might be overcome through additional implementation strategies, more work regarding which specific strategies as well as their feasibility and acceptability within the study clinic are required. Going forward, a research agenda that utilizes a structured approach to developing implementation strategies like the CFIR-ERIC matching tool could help identify the most pragmatic next steps for KSADS-PL implementation at MNH [26].

To the best our knowledge, this study was the first to assess providers’ views about facilitators, barriers and possible solutions to the use of KSADS-PL in Tanzania. A sub-Saharan African based study by Olatunji et al. (2023) highlighted various obstacles faced by children with ADHD in sub-Saharan Africa, focusing on the lack of awareness and stigma surrounding the disorder, limited access to mental health services, and inadequate education and support as barriers to ADHD diagnosis and treatment [27]. The review article suggests public awareness campaigns, training for healthcare professionals, integration of mental health services, addressing affordability and accessibility, telemedicine and digital health platforms, and stakeholder collaboration as useful ways to address the barriers. Similar barriers like lack of awareness to ADHD, limited number of trained mental health providers, and limited material and human resources comprise the results of our current study. Interviewees proposed similar solutions like awareness campaigns, training providers, making services available and accessible, involvement of multiple stakeholders, and use of online assessment methods. However, due to differences in health systems between nations, and the unique nature of each clinic or hospital, preliminary qualitative efforts to better understand unique barriers in each setting are required before implementing wholesale solutions.

Our study findings should be understood within the context of a number of limitations. First, our study was conducted at the only child and adolescent clinic in the country, potentially leaving out some of the barriers and facilitators to the introduction of KSADS-PL into child and adolescent facilities in Tanzania more widely. Nevertheless, as providers at the only tertiary mental health facility in the country, they retain a high level of influence on how screening and diagnosis of child and adolescent mental health disorders might operate over other facilities throughout the country. Secondly, although providers reported that introducing KSADS-PL as feasible with suggested solutions to the identified barriers, we feel that this suggestion is theoretical and requires testing and evaluation.

## Conclusion

Providers reported high levels of feasibility for the KSADS-PL and expressed a need for such a tool on behalf of their clients. At the same time, they identified several barriers relating to human and material resources associated with costs that MNH may struggle to provide (e.g., training and ongoing supervision). While researcher from the region have called for large scale task-shifting efforts to address the dearth of psychiatric service providers [28], several systematic reviews have shown that task-shifting alone is not sufficient to bridge the mental health disorder treatment gap in LMICs, suggesting a need for context-specific adaptations for an implementation strategy to be applied successfully [26].

Literature on implementation strategies is bolstered by research efforts into the barriers and facilitators related to their implementation across contexts [14]. The current study adds to the knowledge base regarding barriers and facilitators to the uptake of KSADS-PL at a large national hospital in Tanzania. While our study represents an important first step in highlighting the feasibility of KSADS-PL implementation in this setting, subsequent research focused on addressing additional barriers like provider workload, turnover, and inadequate materials are still required on the path towards scale-up.

## Supporting information

S1 File Appendix. (DOCX)

S2 File Interview guide: The interview questions to health care provider. (DOCX)
