# Supplementary material for: Barriers, facilitators and potential solutions to implementing Kiddie Schedule for Affective Disorder and Schizophrenia (KSADS) screening tool at Muhimbili National Hospital in Dar es Salaam, Tanzania
Source: PLoS One. 2025 May 9;20(5):e0323502. doi: 10.1371/journal.pone.0323502 (PMC12063892; doi:10.1371/journal.pone.0323502)
Supplement: S1 File — (DOCX) [file pone.0323502.s001.docx]

**S1 File: Appendix**

RESEARCH ARTICLE

1. **Title**: Barriers, facilitators and potential solutions to implementing Kiddie Schedule for Affective Disorder and Schizophrenia (KSADS) screening tool at Muhimbili National Hospital in Dar es Salaam, Tanzania
2. **Authors**

**Leonida I. Ngongi^1^*,** **Christopher F. Akiba^3^, Mrema N. Kilonzo ^2^, Anna A. Minja^2^, Charles O. Komba^1^, Mwajabu R. Mbaga^1^, Anna C. Msafiri^1^, Lusajo J. Kajula^2,4^, Sylvia F. Kaaya ^2^, Brian W. Pence^4^ and Bradley N. Gaynes^4^**

1 Muhimbili National Hospital. 2. Muhimbili University of Health and Allied Science 3. RTI International, Research Triangle Park, NC, USA, 4. Gillings School of Global and Public Health, University of North Carolina At Chapel Hill, Chapel Hill, NC, USA.

*[leonidangongi@yahoo.co.uk](mailto:leonidangongi@yahoo.co.uk)

1. **Key words**: ADHD, children, KSADS-PL, implementation, barrier
2. **Abbreviations**

**ADHD**: Attention Deficit Hyperactivity Disorder

**CFIR**: Consolidated Framework for Implementation Research

**ERIC**: Expert Recommendations for Implementing Change compilation

**DALYS**: Disability Adjusted Life Years

**DSM**: Diagnostic Statistical Manual; MNH: Muhimbili National Hospital

**KSDAS-PL**: Kiddie Schedule for Affective Disorder and Schizophrenia- Life Time version

**IDI**: In‑depth interviews

**IRB**: Institutional Review Boards

**LMIC**: Low and middle‑income countries

**SHARP**: Sub Saharan Africa Capacity Building in Mental Health Research Program

**STEM**: Support, Train and Empower Managers

**MoH**: Ministry of Health

**MNH**: Muhimbili National Hospital

**MUHAS**: Muhimbili University of Health and Allied Sciences and Ministry of Health

1. **Authors’ information**

LIN works as a psychiatrist at MNH, a SHARP pilot grantee, got a pilot grant as principal investigator for conducting this study; CFA works as a researcher at RTI International, Research Triangle Park, NC, USA, 4; MIK is a lecturer, clinical psychologist and researcher at MUHAS; AAM is a qualitative researcher and a social scientist at MUHAS, with post-graduate qualification and previous experience in conducting interviews in the mental health care context; COK, MRM, ACM are medical specialists at MNH; SFK is a professor of psychiatry and mental health at MUHAS; LJK, BNG and BWP are all professors at the University of North Carolina at Chapel Hill.

1. **Funding**

The study received funding from SHARP, funded by the National Institute of Mental Health of the National Institutes of Health (PI: Brian Pence, PhD: U19MH113202‑01). The funders were not involved in the implementation steps of this study.

1. **Data availability**

All relevant data are within the paper and its Supporting Information file.

1. **Ethics approval and consent to participate**

The current study was approved by MUHAS Senate Research and Publications Committee review board approved this pilot study (Ref: No. 282/298/01.C/).

1. **Consent for publication**

As stated on design section above, permission was sought from MNH as the study hospital through hospital director and MNH research unit. All study participants provided written informed consent and waiver to publish data while maintaining their confidentiality.

1. **Competing interests**

The authors declare that they have no competing interests.
