# Supplementary material for: Barriers, facilitators and potential solutions to implementing Kiddie Schedule for Affective Disorder and Schizophrenia (KSADS) screening tool at Muhimbili National Hospital in Dar es Salaam, Tanzania
Source: PLoS One. 2025 May 9;20(5):e0323502. doi: 10.1371/journal.pone.0323502 (PMC12063892; doi:10.1371/journal.pone.0323502)
Supplement: S2 File — (DOCX) [file pone.0323502.s002.docx]

**S2 File: Interview guide: The interview questions to health care provider**

We are introducing K-SADS (a 23 items interview) for screening and diagnosing ADHD in your workplace: -

1. How motivated do you and your colleagues feel about the clinical work that you are doing? What would help motivate you and your colleagues further? (Motivation)
2. Who or what organizations (formal and informal) provide support or not in our communities for children with ADHD or their caregivers/parents? (PROMPT: Tell me more (who, what where, when). How do the organizations/people help your clients? PROBE: Give specific examples and how this makes your work smoother; Do these organizations/people pose challenges to your work? PROBE: Give specific examples
3. Currently how is Attention-Deficit Hyperactivity Disorder (ADHD) being screened and diagnosed in your hospital?

PROBES IF POSITIVE RESPONSE:

- 1. Has it ever been done differently?
  2. What are the reasons of the chosen method of screening and diagnosing?
  3. Are there any other plans for screening and diagnosing ADHD differently in the near future?

PROBES IF NEGATIVE RESPONSE:

- 1. Should health care providers be able to screen and diagnose children with ADHD? If yes, at what level? PROBES Who, where, why should they be able todo?
  2. What needs to happen for providers at your facility to be able to screen and diagnose children with ADHD?

1. ADHD is recommended to be diagnosed by the use of assessment tools, have you ever used them? Could you share your experience on using them?
   1. What type of assessment tools have you ever used? PROBES: Please share examples.
   2. How comfortable were you when using them?
   3. What is easy or difficult about using these tools? PROBES: Please share examples of both ease and difficulty.
2. How well prepared do you feel to provide K-SADS? PROBES Barriers, facilitators, supports needed FOR EACH REQUEST AN EXAMPLE
   1. What are the possible reasons which can hinder your use of K-SADS?
   2. What factors can facilitate or support your readiness to use K-SADS?
3. In intending to use K-SADS in your clinic, an implementation strategy is going to be applied. This is called the educate strategy, and involves in-service training using a one-to-one approach using an iterative strategy between the trainee and trainer, with observation and feed-back with ongoing supportive supervision.
   1. What might be the possible barriers (Barriers are things/measures that hinder the implementation process for K-SADs use) and facilitators (facilitators are things/measures that can help the implementation process for K-SADs use to be accomplished smoothly)
   2. PROBES: for each barrier e.g. B1 XXXX describe to me an example of how this is a barrier and how it affects/may affect K-SADs implementation
   3. PROBES: for each facilitator e.g. F1 XXXX describe to me an example of how this is a facilitator an how it affects/may affect K-SADs implementation
4. In intending to use K-SADS in your clinic, what additional supports do you think you will need? FOR EACH MENTIONED PROBE What makes you say so, what can be done to achieve the needed support?
